# Supplementary material for: MetaRibo-Seq measures translation in microbiomes
Source: Nat Commun. 2020 Jun 29;11:3268. doi: 10.1038/s41467-020-17081-z (PMC7324362; doi:10.1038/s41467-020-17081-z)
Supplement: Supplementary file 10 — Supplementary Data 7 [file 41467_2020_17081_MOESM10_ESM.zip › File2/Confidence_VeryHigh_Taxonomy/146342_out.krona.html]

Javascript must be enabled to view this page.

members
magnitude
magnitudeUnassigned
count
unassigned
taxon
rank

146342\_out

6

superkingdom
6
2

phylum
4
1239

4
class
186801

186802
order
4

1897049

SRS143895\_contig\_number\_25245SRS148196\_contig\_number\_16025SRS893292\_contig\_number\_2862
3
species

186803
1
family

1
species
1898203

SRS893300\_contig\_number\_9788

1224
phylum
2

class
2
28211

order
2
204457

41297
family
2

150203
genus
2

2217673

SRS098073\_contig\_number\_5504SRS149325\_contig\_number\_1272
2
species
